# Left middle lobectomy for bronchiectasis in a patient with Kartagener syndrome: a case report

**DOI:** 10.1186/s13019-016-0426-y

**Published:** 2016-03-09

**Authors:** Haiping Lin, Ziang Cao, Xiaojing Zhao, Qing Ye

**Affiliations:** Department of Thoracic Surgery, Ren Ji hospital, School of Medicine, Shanghai Jiao Tong University, Shanghai, 200127 China

**Keywords:** Kartagener syndrome, Bronchiectasis, Lobectomy

## Abstract

**Background:**

Kartagener syndrome (KS) is a rare disorder characterized by the triad of chronic sinusitis, bronchiectasis, and situs inversus.

**Case presentation:**

A 23-year-old man was admitted to our hospital because of recurrent cough with purulent expectoration, which had occurred intermittently for the past ten years. During the past 3 years, the episode frequency was 3–4 times per year. He was diagnosed with pulmonary infection and bronchiectasis of the left upper lobe, situs inversus, and KS. We concluded that the damaged left middle lobe was the source of repeat pulmonary infections. Thus the left middle lobe resection was performed to remove the source of the lung infection.

**Conclusions:**

The post-operative course was successful and pneumonia was apparently resolved during the 6 months’ follow-up period. We further describe this case in the following report.

## Background

Kartagener syndrome (KS) is a rare recessive autosomal disease with an incidence of approximately 1 in 32,000 live births [1, 2]. It is characterized by primary ciliary dyskinesia accompanied by sinusitis, bronchiectasis, and situs inversus [3]. Our case was diagnosed with synchronous Kartagener syndrome, pulmonary infection and bronchiectasis of the left upper lobe. Here, we reported the left middle lobectomy for a KS patient with bronchiectasis, which has been rarely reported in the literature.

## Case presentation

A 23-year-old unmarried man was admitted to the Department of thoracic surgery of Renji hospital (Shanghai, China) because of recurrent cough with purulent expectoration, which had occurred intermittently for the past ten years. During the past 3 years, the episode frequency was 3–4 times per year, and an increased frequency occurred over the past 6 months. He had seen a respiratory physician many times and was diagnosed with pneumonia due to bronchiectasis. Additionally, he had received several courses of intravenous antibiotic therapy over the past 6 months, however, frequent relapse occurred after antibiotic therapy. He had no fever, dyspnea and hemoptysis. Further questioning revealed that the patient used to suffer from sinusitis since his teenage years, presenting symptoms including repeated nasal congestion and nasal discharge. The patient acknowledged that he had been diagnosed with dextrocardia in childhood. He had no brothers or sisters, and his parents were healthy.

Yellowish discharge could be observed in the nasal cavity without obvious press-pain on paranasal sinus areas. The thoracic wall had no deformities. Chest auscultation revealed crepitations in the left infrascapular region and normal heart sounds on the right side. The apex beat was palpable over the fifth intercostal space on the right side of chest. The other physical and systemic examinations were normal.

**Fig. 1:**
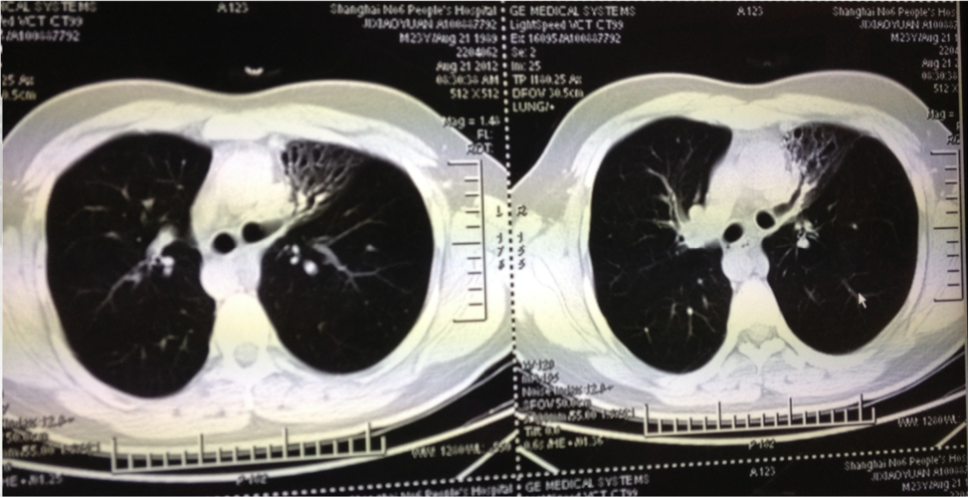
Chest computed tomography scan showing bronchiectasis in the local left lung

**Fig. 2:**
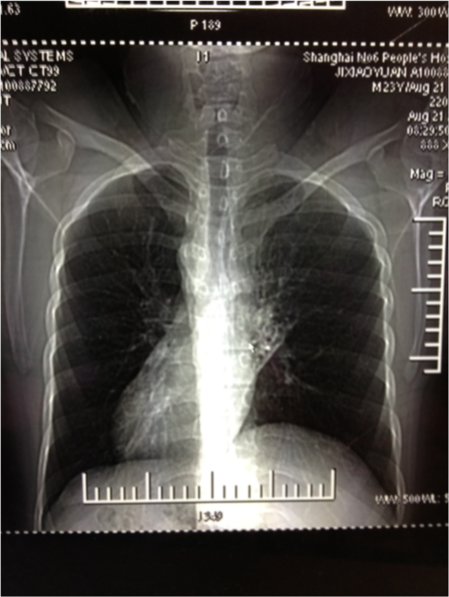
Chest computed tomography scan showing dextrocardia

Hemogram revealed a total leukocyte count of 11.0 × 10^9^/L (normal range: 3.97–9.15 × 10^9^/L), a neutrophil percentage of 78.5% (normal range: 50–70%), and a hemoglobin of 150 g/L (normal range: 131–172 g/L). Liver functions and renal functions were normal, and the erythrocyte sedimentation rate was 40 mm/H. Sputum smear examination for *Mycobacterium tuberculosis* was negative, and the blood tuberculosis antibody was also negative. Abdominal ultrasound revealed complete situs inversus and the organs were normal. Chest computed tomography (CT) scan revealed a bronchiectasic appearance in the local lung tissue of the left middle lobe (Fig. 1) and dextrocardia (Fig. 2). His admission diagnosis included pulmonary infection and bronchiectasis of the left middle lobe, situs inversus, and possible KS.

**Fig. 3:**
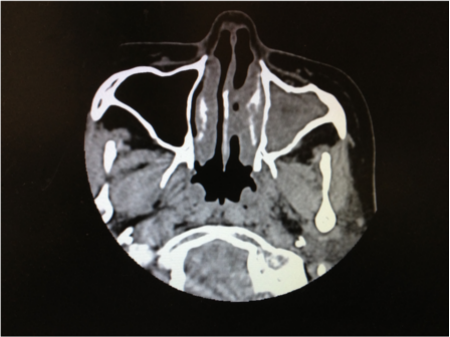
Chest computed tomography scan showing left ethmoidal and maxillary sinusitis

Paranasal sinus CT scan revealed that his nasal mucosa obviously thickened, besides, his left maxillary sinus and ethmoidal sinus mucosa swelled obviously (Fig. 3). The Kartagener triad of symptoms was observed in this patient, which confirmed the diagnosis of KS. His primary problem was recurrent pneumonia due to bronchiectasis, which had to be resolved effectively. After careful examination of the chest CT, we concluded that the bronchiectasic lung was destroyed and had clear boundaries. Regarding his situs inversus, the left lung was clearly composed of three lobes, with lesion in the left middle lobe. There was no bronchiectasic signs in the upper or lower left lobe or in the right lung. Thus, we concluded that the damaged left middle lobe was the source of repeat pulmonary infections. Altogether, these results suggested that this lobe had to be resected, as the infections would otherwise not be curable.

**Fig. 4:**
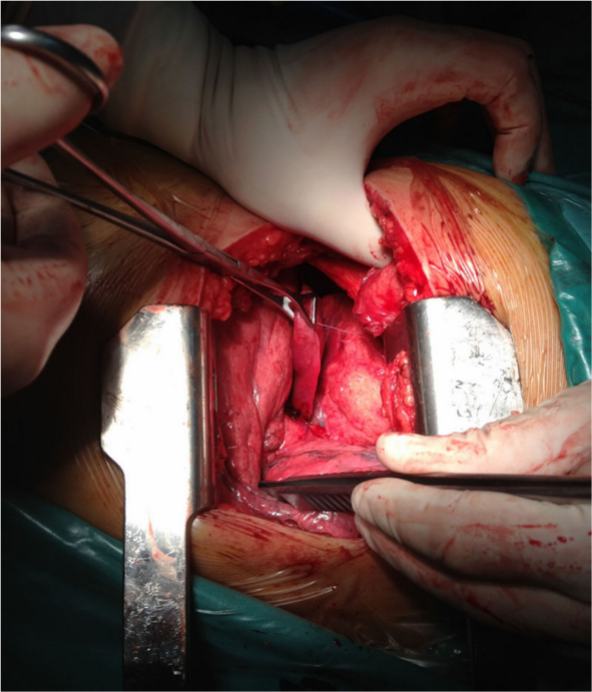
Consolidation of the left middle lobe (held by lung-grasping forceps)

**Fig. 5:**
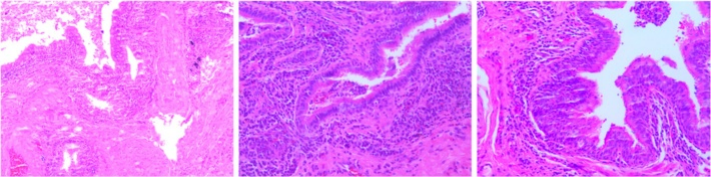
The histologic images of the resected left middle lobe. A: 100 ×; B and C: 200×

**Fig. 6:**
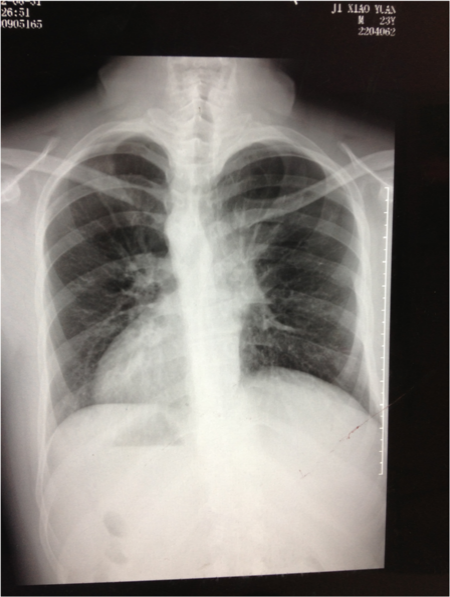
Chest X-rays showing recovery after six months follow-up

After three days of antibiotic treatment (2 g cefotiam through an intravenous drip), left thoracotomy was performed via left posterolateral incision under the condition of general anesthesia. The fifth intercostal muscles were incised layer by layer, and sixth rib was cut off and opened with a rib retractor. The double lumen endotracheal catheter (Covidien Mallinckrodt Endobronchial Tube 37Fr left; Covidien, USA) was intubated into the left main bronchus. It was confirmed that the patient’s left lung had three lobes, and the azygos vein and superior vein cava could be exposed in the left thoracic cavity, confirming situs inversus. The middle lobe was collapsed and consolidated (Fig. 4), although the upper and lower lobes seemed normal and the fissures had completely developed. Before the left middle lobe was removed, the middle lobe branch of the pulmonary artery and vein, and the bronchus of the middle lobe were excised and sutured individually with staples (Covidien, USA). After lobe was resected, wound surface was stanched and the chest was washed with physiological saline water for 3 times. The flushing fluid was removed and lung wound surface was sutured with 3-0 Prolene. Rib incision was sutured with No. 10 silk; muscle layers were sutured with 2-0 Johnson ETHICON Vicryl; skin was sutured with 3-0 Johnson ETHICON Vicryl. In addition, a 28F chest tube was placed post-operatively and was removed 3 days later. The patient-controlled intravenous analgesia (PCIA) with sufentanil 0.2 mg combined with granisetron 3 mg was provided for pain relief for 48 h. The operation was completed without complication. The final pathology examination (Fig. 5) revealed the occurrence of bronchiectasis and hyperemia in this lobe, as well as the proliferation of lymphocytes and plasmocytes around the bronchus. The post-operative antibiotic therapy (2 g cefotiam through an intravenous drip) lasted 7 days, and chest CT indicated left lung recovered well without pulmonary infection. On postoperative day 8, the patient was discharged. Regular follow-up was conducted by the ear-nose-throat (ENT) specialist and thoracic surgeons. Mucolytics, such as myrtol standardized capsules (Pohl-Boskamp GmbH & Co. KG, Germany; 0.3 g three times a day), was taken to treat his remaining sinusitis, as administered by the ENT doctors. After 6 months of follow-up, his symptoms of cough and purulent sputum were relieved, and his nasal congestion and discharge were alleviated. Follow-up chest X-rays confirmed no further issues (Fig. 6).

## Discussion

KS patients generally present with recurrent lower respiratory tract infection and nasal sinusitis due to ineffective mucociliary clearance [4]. Additionally, male patients are usually sterile, as sperm motility is dependent on ciliary function [5]. In the present study, testing in primary ciliary dyskinesia was not performed because of the technical condition constraints in our hospital, which was a limitation of our study. Importantly, the triad of symptoms in KS was observed in the present case, although no male infertility was noted because sperm motility was not assessed.

The left middle lobectomy in KS has rarely been reported due to the rarity of this disorder. The present case recovered well after the left middle lobectomy. We therefore summarized the treatment method for this disease.

The primary symptom of the patient was recurrent respiratory tract infection owing to bronchiectasis in the left middle lobe. The damaged lung was the source of the irreversible infection, if untreated, the range of the lesion would expand to further destroy the lung tissue. It has been reported that pulmonary heart disease may occur if lung transplantation is not performed in an end-stage KS patient [6–8]. Therefore, the left lobe resection was necessary in this case. Specially, the advantage of pulmonary resection for treating chronic bronchiectasis has been reported [9]. During the follow-up, the respiratory tract infection did not occur any further, although the patient suffered from occasional nasal congestion and discharge due to the ineffective mucociliary clearance. The results of the left lobectomy were desirable for him. However, the patient should continue to see his ENT specialist for the clinical treatment of sinusitis. Importantly, myrtol could help alleviate his symptoms.

## Conclusions

In conclusion, serious damage of the lung may occur after bronchiectasis and pneumonia in KS patients unless a diagnosis of this rare syndrome can be confirmed and therapy is provided. Surgical resection is an acceptable method for patients whose bronchiectasic lungs are localized within one or two lobes. To the best of our knowledge, we are the first to report left middle lobectomy for treating bronchiectasis in KS patient. The progression of bronchiectasis was halted by surgery during the immediate post-operative follow-up period. For this reason, it should be emphasized that KS requires a high degree of suspicion for its early diagnosis by physicians, ENT surgeons, and thoracic surgeons.

## Consent

Written informed consent was obtained from the patient for publication of this Case report and any accompanying images. A copy of the written consent is available for review by the Editor of this journal.

## Highlights

1. Chest CT revealed bronchiectasis inthe left upper lobe and dextrocardia.
2. Paranasal sinus CT scan revealed left ethmoidal and maxillary sinusitis.
3. The left middle lobectomy was performed and the patient recovered well after surgery.

## Abbreviations

CT: computed tomography
ENT: ear-nose-throat
KS: Kartagener syndrome
